# RETRACTED: Sharad et al. Analysis of *PMEPA1* Isoforms (*a* and *b*) as Selective Inhibitors of Androgen and TGF-β Signaling Reveals Distinct Biological and Prognostic Features in Prostate Cancer. *Cancers* 2019, *11*, 1995

**DOI:** 10.3390/cancers18050825

**Published:** 2026-03-04

**Authors:** Shashwat Sharad, Zsófia M. Sztupinszki, Yongmei Chen, Claire Kuo, Lakshmi Ravindranath, Zoltan Szallasi, Gyorgy Petrovics, Taduru L. Sreenath, Albert Dobi, Inger L. Rosner, Alagarsamy Srinivasan, Shiv Srivastava, Jennifer Cullen, Hua Li

**Affiliations:** 1 Center for Prostate Disease Research, Department of Surgery, Uniformed Services University of the Health Sciences and the Walter Reed National Military Medical Center, 6720A Rockledge Drive, Suite 300, Bethesda, MD 20817, USA; cymclg@gmail.com (Y.C.); ckuo@cpdr.org (C.K.); lravindranath@cpdr.org (L.R.); gpetrovics@cpdr.org (G.P.); tsreenath@cpdr.org (T.L.S.); adobi@cpdr.org (A.D.); inger.l.rosner.mil@mail.mil (I.L.R.); alagarsamy.srinivasan@gmail.com (A.S.); shsr629@gmail.com (S.S.); jcullen@cpdr.org (J.C.); 2 John P. Murtha Cancer Center, Walter Reed National Military Medical Center, Bethesda, MD 20817, USA; 3 Henry Jackson Foundation for the Advancement of Military Medicine, 6720A Rockledge Dr, Suite 100, Bethesda, MD 20817, USA; 4 Danish Cancer Society Research Center, 2100 Copenhagen, Denmark; sztup@hotmail.com (Z.M.S.); zoltan.szallasi@childrens.harvard.edu (Z.S.); 5 Computational Health Informatics Program, Boston Children’s Hospital, Harvard Medical School, Boston, MA 02115, USA; 6 SE-NAP Brain Metastasis Research Group, 2nd Department of Pathology, Semmelweis University, 1085 Budapest, Hungary; 7 Department of Urology, Walter Reed National Military Medical Center, Bethesda, MD 20814, USA

The journal retracts the article titled “Analysis of PMEPA1 Isoforms (a and b) as Selective Inhibitors of Androgen and TGF-β Signaling Reveals Distinct Biological and Prognostic Features in Prostate Cancer” [1], cited above.

Following publication, concerns were brought to the attention of the Editorial Office regarding the integrity of several images presented in this publication [1].

Adhering to our standard journal procedure, the Editorial Office and Editorial Board investigated, with input from a relevant institution. The investigation confirmed a partial duplication between Figure 4B [1] and Figure 4C [2] and a duplication between Figure 4H or Figure 5I in [1] and Figure 4H or Figure 6I in [2], produced by a similar authorship group, presenting different experimental conditions. The authors did not provide a response to the concerns raised. A report provided by the institution confirmed the above-mentioned issues and clarified that that original material was not available for Editorial Board evaluation.

Consequently, the Editorial Board has lost confidence in the reliability of the data and findings and has decided to retract this publication [1], as per MDPI’s retraction policy (https://www.mdpi.com/ethics#_bookmark30).

This retraction was approved by the Editor-in-Chief of the journal *Cancers*.

The authors did not provide any comments on this decision.
